# The Biological Characteristics of Novel H5N6 Highly Pathogenic Avian Influenza Virus and Its Pathogenesis in Ducks

**DOI:** 10.3389/fmicb.2021.628545

**Published:** 2021-01-26

**Authors:** Jianni Huang, Siyu Wu, Wenbo Wu, Yiwen Liang, Haibin Zhuang, Zhiyu Ye, Xiaoyun Qu, Ming Liao, Peirong Jiao

**Affiliations:** ^1^Department of Animal Infectious Diseases, College of Veterinary Medicine, South China Agricultural University, Guangzhou, China; ^2^Guangdong Laboratory for Lingnan Modern Agriculture, Guangzhou, China; ^3^Key Laboratory of Zoonoses Prevention and Control of Guangdong Province, Guangzhou, China; ^4^Pearl River Fisheries Research Institute, Chinese Academy of Fishery Sciences, Guangzhou, China

**Keywords:** H5N6 avian influenza virus, genetic evolution, pathogenicity, transmission, duck

## Abstract

Clade 2.3.4.4 H5Nx highly pathogenic avian influenza viruses (HPAIVs) have caused outbreaks in poultry in the world. Some of these viruses acquired internal genes from other subtype avian influenza viruses (AIVs) such as H9 and H6 for the generation of novel reassortant viruses and continually circulated in poultry. Here, we applied a duck-origin virus DK87 and a chicken-origin virus CK66 to assess the biological characteristics of novel reassortant H5N6 HPAIVs and its pathogenesis in ducks. A genetic analysis indicated that the HA genes of the two H5N6 HPAIVs were closely related to the H5 viruses of clade 2.3.4.4 circulating in Eastern Asia and classified into H5 AIV/Eastern Asia (EA)-like lineage. Their NA genes fell into Eurasian lineage had close relationship with those of H5N6 viruses circulating in China, Laos, Vietnam, Japan, and Korea. All internal genes of DK87 were aggregated closely with H5 AIV/EA-like viruses. The internal genes (PB1, PA, NP, M, and NS) of CK66 were derived from H9N2 AIV/SH98-like viruses and the PB2 were derived from H5 AIV/EA-like viruses. These results indicate that clade 2.3.4.4 H5N6 AIVs have continually evolved and recombined with the H9N2 viruses circulating in Southern China. Pathogenicity test showed that the two viruses displayed a broader tissue distribution in ducks and caused no clinical signs. These results indicated that ducks were permissive for the replication of the chicken-origin reassortant virus CK66 without prior adaptation, but the duck-origin virus DK87-inoculated ducks showed significantly higher viral titers in some organs than the CK66-inoculated ducks at 5 day post-inoculated (DPI). The recovery of viruses from oropharyngea and cloacal swabs of contacted ducks indicated that they transmitted in native ducks by direct contact. Quantitative reverse transcription PCR (qRT-PCR) results revealed that the immune-relative genes (PRRs, IFNs, Mx-1, IL-6, and IL-8) in the lungs of inoculated ducks were expressed regardless of virus origin, but the expression of these genes was significantly higher in response to infection with the DK87 virus compared to the CK66 virus at 3 DPI. Overall, we should provide further insights into how clade 2.3.4.4 H5N6 AIVs undergo genetic and pathogenic variations to prevent outbreaks of this disease.

## Introduction

Avian influenza viruses (AIVs) are enveloped, segmented, single-stranded negative sense RNA viruses belonging to the family *Orthomyxoviridae* (Swayne and Suarez, 2000). AIVs are highly pathogenic viruses (HPAIVs) and low pathogenic avian influenza viruses (LPAIVs) according to their pathogenicity in domestic chickens. However, most outbreaks are caused by H5 or H7 subtype HPAIVs (Swayne and Suarez, 2000).

Since the H5N1 HPAIVs were first isolated in China in 1996, H5 subtype viruses have evolved into multiple clades from clade 0 to clade 9. With growing number of isolates, clade 2 H5 subtype AIVs have expanded into second- (e.g., clade 2.1) and third-order (e.g., clade 2.3.4) clades during 2006–2008. Those clades were then further expanded into additional fourth-order clades (e.g., clade 2.3.4.4; Gu et al., 2013). Since 2008, H5 HPAIVs bearing the genetic backbone of clade 2.3.4.4 H5N1 have been identified in China and evolved into different subtypes including H5N1, H5N2, H5N5, H5N6, and H5N8 by acquiring NA gene from other AIVs (Yamaji et al., 2020). Among these reassortments, H5N6 AIVs have become dominantly prevalent subtypes in Southern China especially in waterfowl since 2014 (Bi et al., 2016). From 2014 to 2017, 40 outbreaks of H5N6 AIVs in 16 different regions of China resulting in 72,446 bird deaths have been reported (OIE, 2017). Generally, rapid evolution and circulation of the H5 subtype AIVs of clade 2.3.4.4 pose an increased threat to poultry.

Ducks are a natural reservoir of AIVs and are permissive for replication of most strains. Moreover, ducks can transmit AIVs to other avian species such as terrestrial poultry, which contributes to the circulation of AIVs (Wei et al., 2013). Ducks have been described as “Trojan horses” due to the silent spread of H5N1 HPAIVs without clinical signs. However, ducks infected with H5N1 HPAIVs gradually showed clinical signs ranging from asymptomatic infections to severe disease with mortality (Kim et al., 2009). Previous studies showed that AIVs of clade 2.3.4.4 were highly lethal to chickens but caused a variety of symptoms in ducks inoculated with different viruses (Sun et al., 2016). Therefore, the pathogenicity of clade 2.3.4.4 H5N6 AIVs in ducks should be studied further.

The innate immune response is the host’s first line defense against influenza infection. It is activated *via* the recognition of pathogen-associated molecular patterns (PAMPs, such as lipoproteins, nucleic acids, and pathogen-specific carbohydrates) by pattern recognition receptors (PRRs) leading to the secretion of antiviral cytokines and pro-inflammatory cytokines. The outcome of moderate innate immune response maybe inhibit the AIVs replication, but the excessive pro-inflammatory cytokines (“cytokine storms”) enhance the damage to human, mice, and chickens (Us, 2008). Previous studies indicated that chickens infected with H5N1 AIVs had remarkably high levels of antiviral cytokines (e.g., IFN β and OAS) and pro-inflammatory cytokines (e.g., IL-6), but ducks infected with H5N1 viruses had a lower inflammatory response than in chickens (Wei et al., 2013; Burggraaf et al., 2014). However, only few studies have investigated the host immune response of ducks inoculated the H5N6 AIVs in clade 2.3.4.4.

Here, we described the genetic characteristics of two H5N6 HPAIVs and analyze their pathogenicity and transmission in ducks. We also evaluated the mRNA level of innate immune-related genes in the lungs of ducks infected with the two H5N6 HPAIVs at different time points.

## Materials and Methods

### Viruses

The two H5N6 AIVs in this study, A/chicken/Guangdong/CK66/2016 (CK66) and A/duck/Guangdong/DK87/2016 (DK87), were isolated from cloacal swabs of apparently healthy birds in live bird markets (LPMs) in Guangdong in 2016. The two viruses were propagated and purified in the allantoic cavity of 9-day-old embryonated specific-pathogen-free (SPF) chicken eggs. The allantoic fluid identified positive by hemagglutination test was collected and frozen at −80°C (Chen et al., 2004). The evaluation of 50% egg infective doses (EID_50_) was calculated as described in the Reed-Muench method. All viral experiments were performed in animal biosafety level 3 (ABSL-3) facilities.

### Pathogenicity and Transmission

The 4-week-old Muscovy ducks used in this study were obtained from a farm in Guangdong and raised in the isolators of ABSL-3 facilities. Serum samples were collected from all ducks to confirm that the ducks were serologically negative for avian influenza by hemagglutination inhibition (HI) test before infection. The experiment design has been described previously (Jiao et al., 2018). In brief, ducks (*n*=28) were divided into two groups (DK87-inoculated group and CK66-inoculated group), 14 per group. In each inoculated group, each duck was inoculated intranasally with 0.2ml of 10^7^ EID_50_ of the H5N6 virus (DK87 or CK66), respectively. To study transmission of the two viruses, five uninfected ducks (“contact ducks”) were inoculated intranasally with 0.2ml of phosphate buffered saline (PBS) and were housed in each isolator with the inoculated ducks at 24-h post-inoculated (HPI). The clinical symptoms of all ducks were monitored for 14days or until they died due to virus infection. At 12 HPI, 3- and 5-day post-inoculated (DPI), three ducks in each inoculated group were euthanized to quantitate the viruses in lung, liver, spleen, kidney, brain, trachea, pancreas, intestines, and cloacal bursa, respectively. Similar treatment was performed on ducks including the inoculated and contacted groups that had died. To assess viral shedding in ducks, oropharyngeal and cloacal swabs were taken from ducks at 3, 5, 7, 9, 11, and 13 DPI and suspended in 1ml PBS. All tissues and swabs were collected and titrated for virus infectivity in eggs. The organ samples (1g per tissue) were weighted and homogenized in 1ml ice-cold PBS contained with 1,000U/ml of penicilin and 1,000U/ml of streptomycin. The supernatant fluids was harvested and clarified by centrifugation (4,000×*g* for 10min). Serial 10-fold dilution of the resulting supernatants was prepared in PBS and 100μl of each dilution was inoculated into the allantoic cavity of 9–10-day-old embryonated eggs. The eggs were incubated at 37°C for 48h. Hemagglutination assays were used to detect the virus titers (calculated using the method of Reed and Muench method; Thakur and Fezio, 1981). Serum was collected from all the surviving ducks at 14 DPI, and seroconversion was confirmed by HI test. The data of virus titration are represented by means±SD for three individually infected ducks.

To obtain relative quantitative level of expression of immune-relative genes in the lungs of inoculated ducks, the control group contained six ducks inoculated intranasally with 0.2ml of PBS. Three control ducks were euthanized at 12 HPI and 3 DPI, and their lungs were collected.

### Quantification of Immune-Relative Genes in the Lungs of Inoculated Ducks

To evaluate the immune response induced by the two H5N6 AIVs, total RNA from lungs of inoculated ducks and control ducks were isolated with the Eastep® super total RNA extraction kit following the protocol of the manufacturer (Promega, China). The quality and quantity of RNA in each sample were measured by Uitro-spec 2000 mass spectrophotometer. Extracted RNA samples were treated enzymatically with DNAase I (Takara, Japan) according to the manufacturer’s protocols. Reverse transcription was performed by using a SuperScript III First Strand synthesis system (Life Technologies, United States) in 80μl of reaction mixture, containing 1μg of total RNA at 37°C for 2h. All the harvested cDNA samples were stored at −80°C for further study.

SYBR Green I based real-time PCR was employed using a Bio-Rad CFX96 Touch™ Real-Time PCR Detection System (Bio-Rad Laboratories, United States). Primers for real-time PCR used in this study were designed by Oligo7 software (Molecular Biology Insights Inc., USA). The primers developed for β-actin (EF667345.1) were *F: GATCACAGCCCTGGCACC and R: CGGATTCATCATACTCCTGCTT*, and the primers developed for RIG-I, TLR3, TLR7, MDA5, IL-6, IL-8, IFN ɑ, IFN β, and Mx-1 molecules have been described previously (Wei et al., 2013). The real-time PCR reactions were performed in 96-well plates with a final reaction volume of 20μl. The PCR conditions are as follows: 1cycle of 95°C for 5min followed by 40cycles of 95°C for 15s and 60°C for 34s. The relative quantification of cytokines was calculated according to 2^-ΔΔCt^ methods *via* the house keeping gene β-actin as an internal control to normalize the level of target gene expression. The relative expressions of cytokines in infected ducks were compared with that of the control group.

### Phylogenetic and Sequence Analysis

Eight segments of the two H5N6 AIVs were sequenced. Viral RNA was extracted from allantoic fluid with Trizol LS Reagent (Invitrogen Life Technologies, Inc., United States) that was transcribed to cDNA using the M-MLV Reverse Transcriptase (Promega, China). PCR amplification was performed with specific primers, and PCR products were purified using a DNA Purification Kit (TIANGEN Biotech, China). All PCR products were identified by Shanghai Invitrogen Biotechnology Co., Ltd. DNA sequences were compiled and edited with the SEQMAN program of Lasergene7.1 (DNASTAR, United States). Phylogenetic trees were constructed using the distance-based neighbor-joining method with software MEGA 5 (Sinauer Associates, Inc., United States), *via* the maximum likelihood method with bootstrap analysis (1,000 replicates). Horizontal distances are proportional to genetic distance. The nucleotide sequences obtained in our study have been deposited in GenBank database, under the following accession numbers (MW090832-MW090847).

### Ethics Statement

All experiments involving animals were performed in ABSL-3 facilities and experimental protocols (SCAUABSL2017-019) were approved by the biosafety committee of South China Agriculture University. Housing animals were conducted in accordance with guidelines of experimental animal administration and ethics committee of South China Agriculture University (SCAUABSL2017-019; July 7, 2017).

### Statistical Analysis

Statistically significant differences in avian influenza replication and real-time quantitative PCR (qPCR) data were analyzed by the GraphPad Prism 5.0 software (GraphPad Software Inc., San Diego, CA, United States). The value of *p*<0.05, *p*<0.01 and *p*<0.001 were considered significant, very significant, and extremely significant, respectively.

## Results

### Genetic Characteristic of the Two H5N6 HPAIVs

**Figure 1:**
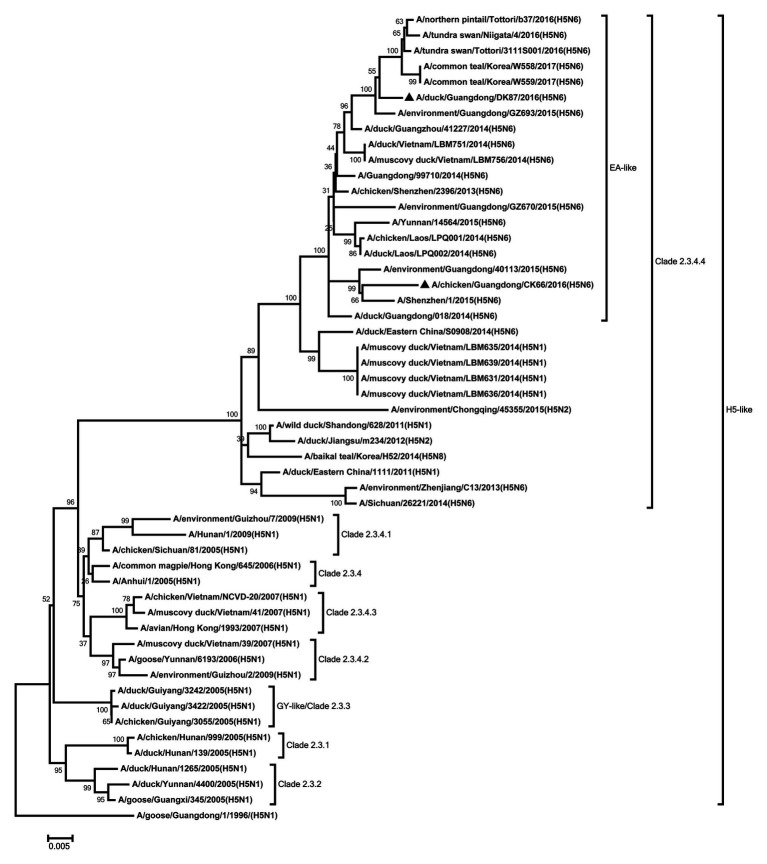
Phylogenetic analysis of HA. HA: nucleotides (nt) 1–1,673. Black triangles indicate viruses characterized in this study; other viral sequences were downloaded from GenBank. GY, Guiyang; EA, Eastern Asia, viruses came from China, Laos, Vietnam, Japan, Korea, and so on. H5, came from H5 subtype AIVs. Virus like/lineages are shown at right.

Eight genes of the two H5N6 HPAIVs were sequenced to analyze the genetic evolution. The HA genes of our viruses were closely related to the H5 viruses of clade 2.3.4.4 circulating in Eastern Asia and classified into H5 AIV/Eastern Asia (EA)-like lineage (Figure 1). Their nucleotide sequence showed a similarity 96.4% between each other. The amino acid sequence of the cleavage site in the HA protein of the DK87 was PLKERRRKR/GLF, and the CK66 virus was PLRERRRKR/GLF, which is characteristic of HPAIV (Supplementary Table S1). The receptor-binding site at the 226–228 (H3 numbering) motif was QQG of DK87 virus and QSG of CK66 virus, which indicated that they preferred binding to avian-like (α2–3-galactose sialic acids) receptors. Both of these viruses exhibited seven potential glycosylation sites at 26, 27, 39, 181, 302, 499, and 558 (H5 numbering; Supplementary Table S2).

**Figure 2:**
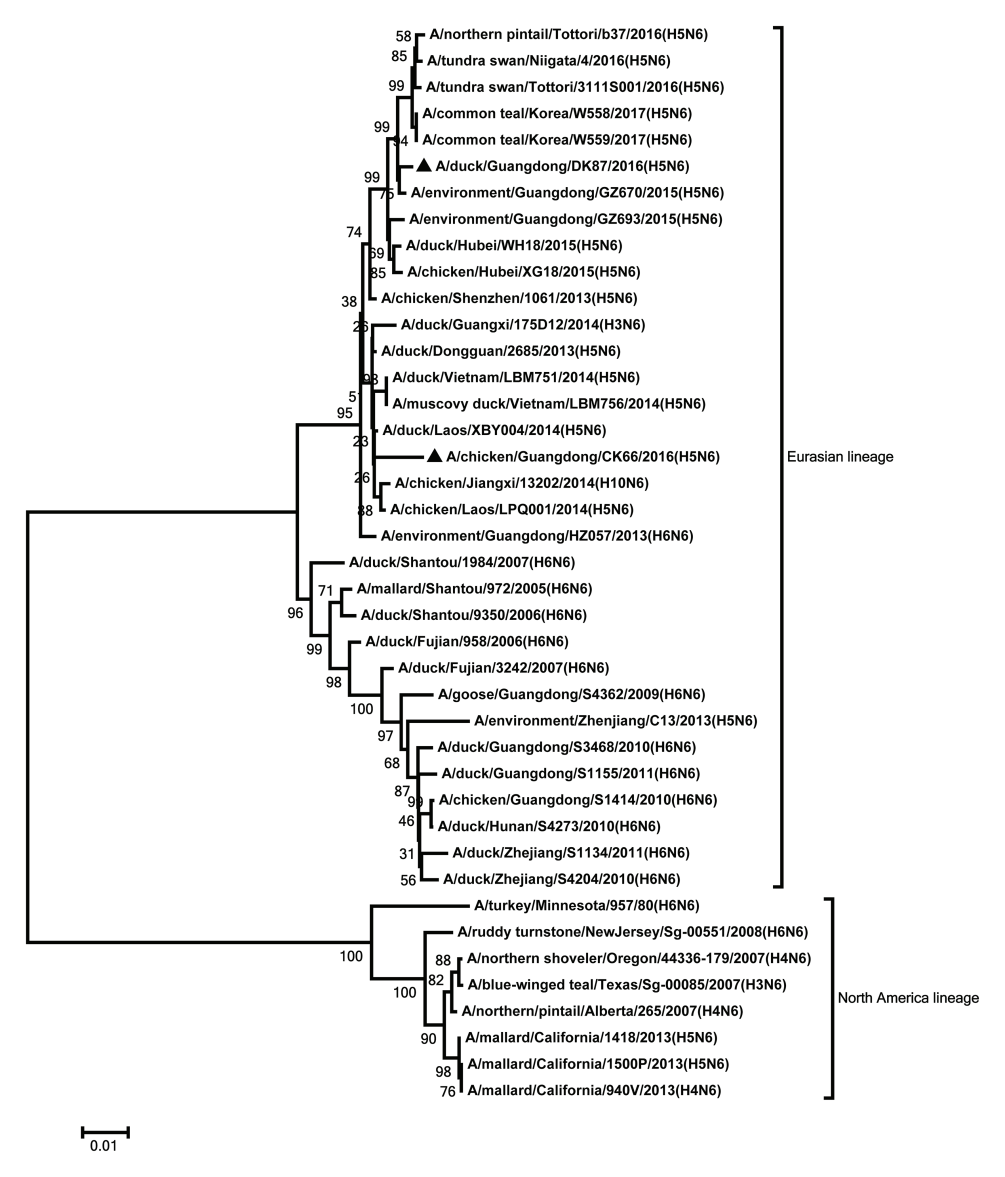
Phylogenetic analysis of NA. NA: nt 1–1,366. Black triangles indicate viruses characterized in this study; other viral sequences were downloaded from GenBank. Virus like/lineages are shown at right.

The NA genes of the two viruses were clustered into Eurasian lineage and had close relationship with those of H5N6 viruses circulating in China, Laos, Vietnam, Japan, and Korea (Figure 2). The two viruses showed 96.2% nucleotide sequence similarity with each other. Deletions in the NA stalk region (11 amino acids in N6, positions 58–68) were observed in both viruses. The two viruses shared six potential glycosylation sites at positions 51, 54, 70, 86, 146, and 201 in the NA gene (Supplementary Table S2).

**Figure 3:**
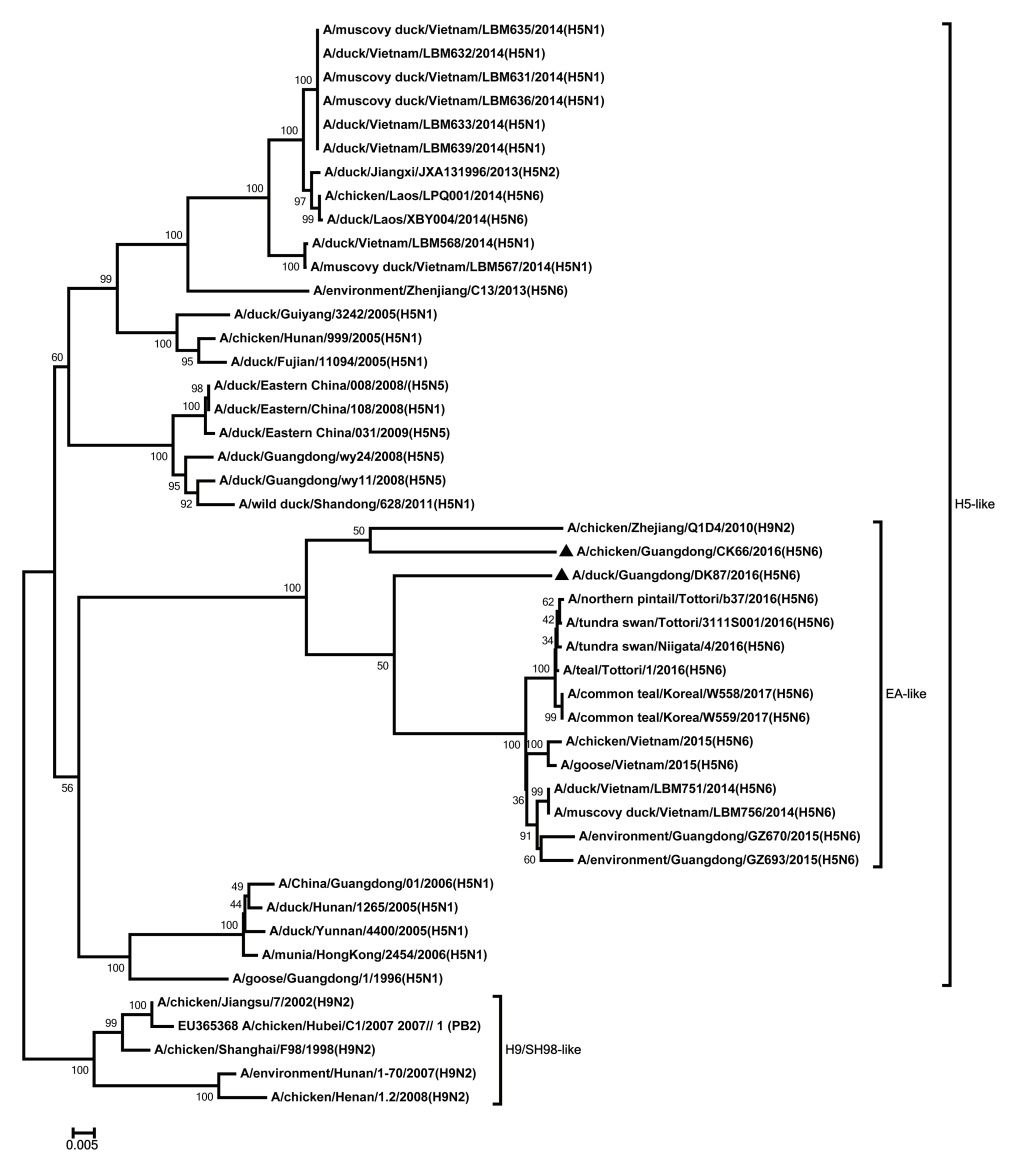
Phylogenetic analysis of PB2. PB2: nt 1–2,254. Black triangles indicate viruses characterized in this study; other viral sequences were downloaded from GenBank. EA, Eastern Asia, viruses came from China, Laos, Vietnam, Japan, Korea, and so on; H5, came from H5 subtype AIVs; H9/SH98, came from H9 subtype AIVs; SH, Shanghai. Virus like/lineages are shown at right.

**Figure 4:**
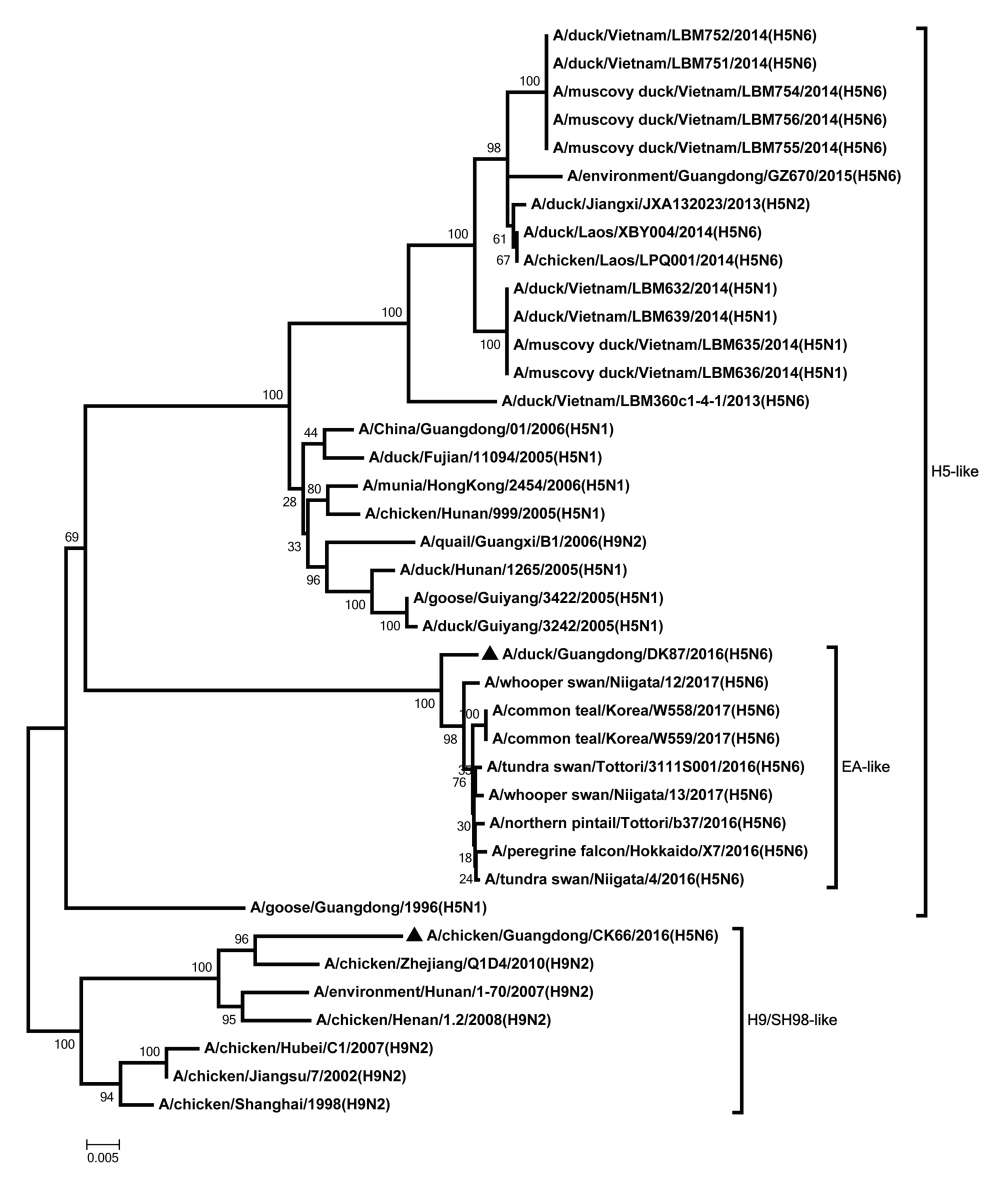
Phylogenetic analysis of PB1. PB1: nt 1–2,209. Black triangles indicate viruses characterized in this study; other viral sequences were downloaded from GenBank. EA, Eastern Asia, viruses came from China, Laos, Vietnam, Japan, Korea, and so on; H5, came from H5 subtype AIVs; H9/SH98, came from H9 subtype AIVs; SH, Shanghai. Virus like/lineages are shown at right.

**Figure 5:**
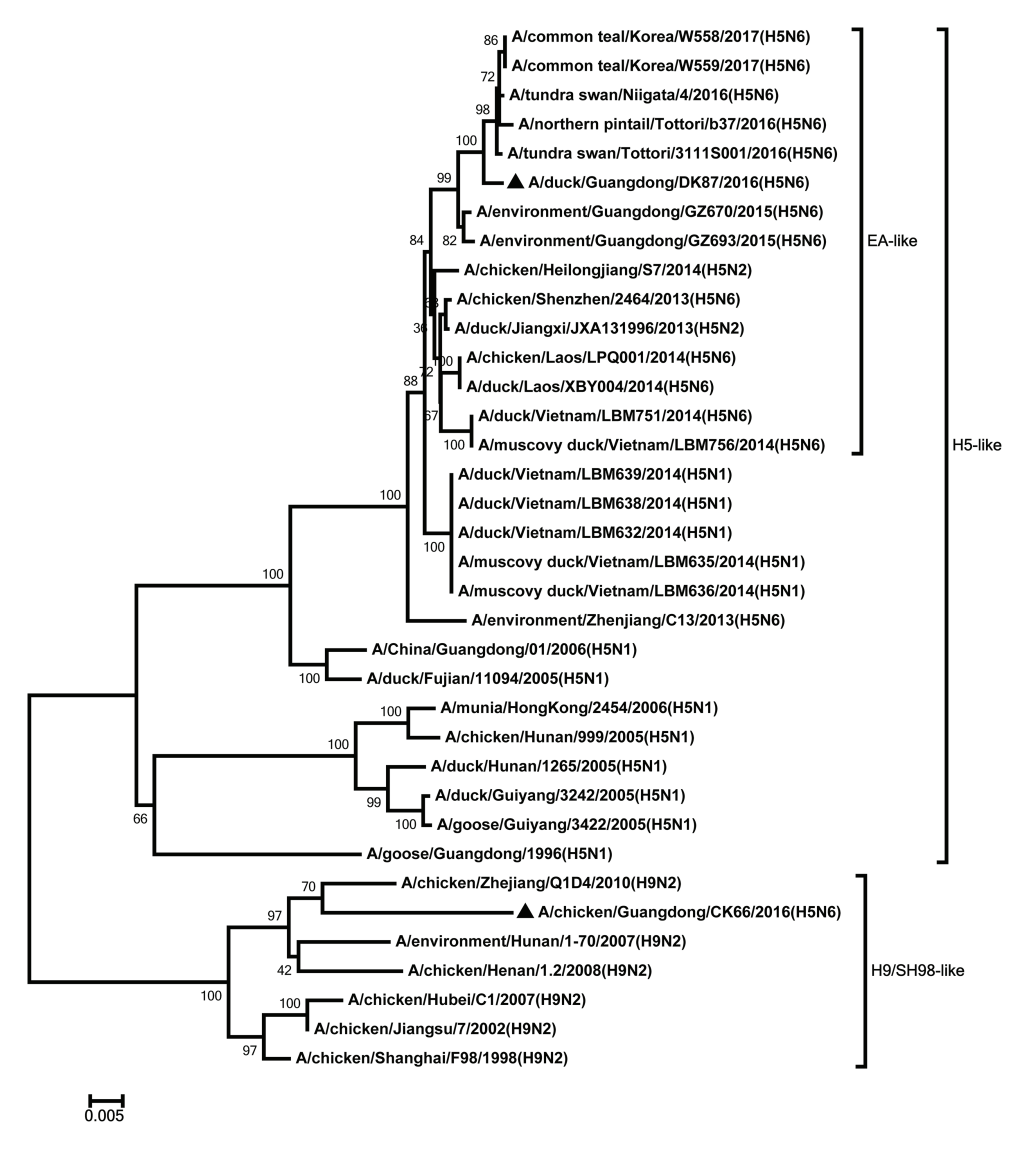
Phylogenetic analysis of PA. PA: nt 1–2,110. Black triangles indicate viruses characterized in this study; other viral sequences were downloaded from GenBank. EA, Eastern Asia, viruses came from China, Laos, Vietnam, Japan, Korea, and so on; H5, came from H5 subtype AIVs; H9/SH98, came from H9 subtype AIVs; SH, Shanghai. Virus like/lineages are shown at right.

**Figure 6:**
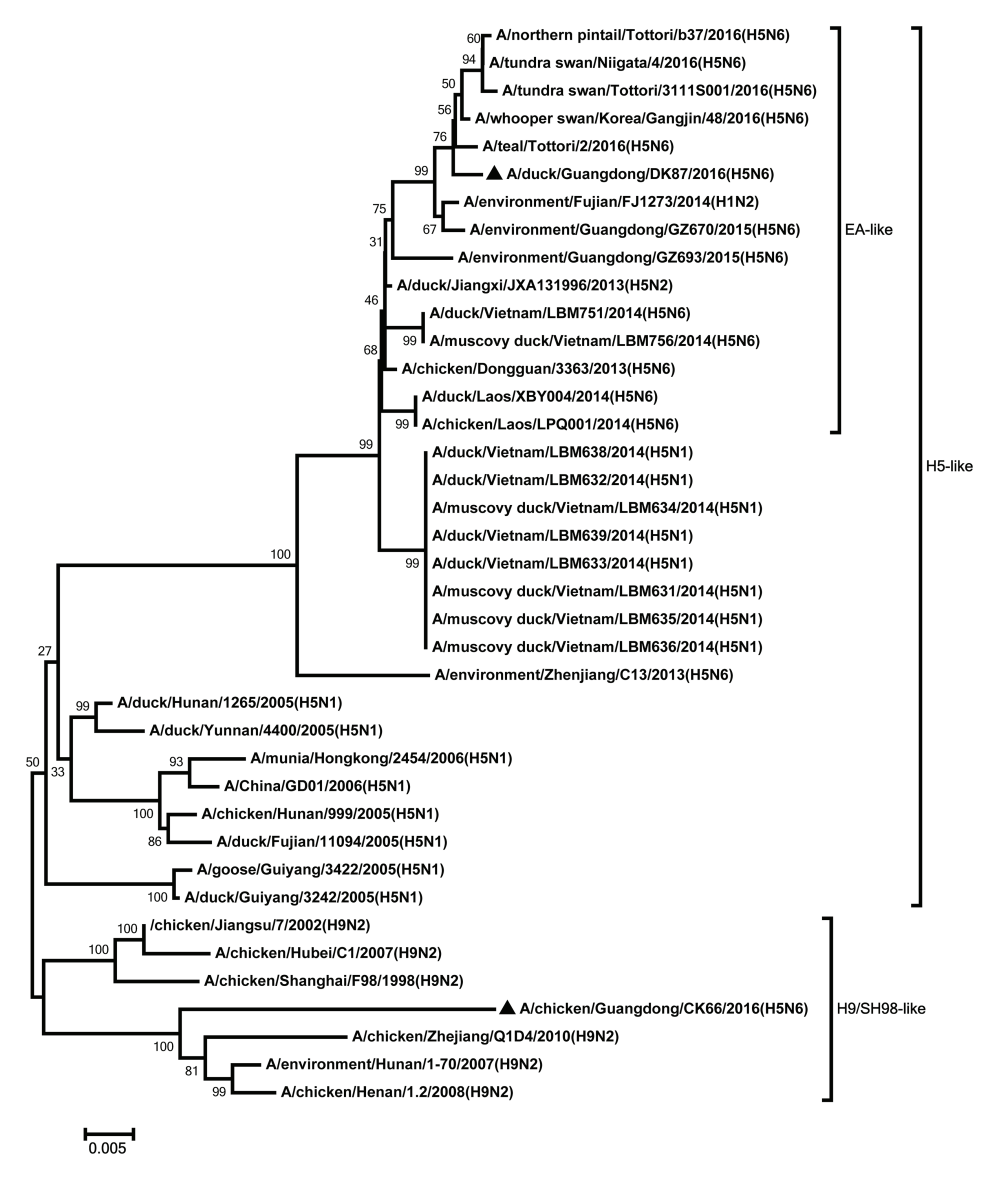
Phylogenetic analysis of NP. NP: nt 1–1,409. Black triangles indicate viruses characterized in this study; other viral sequences were downloaded from GenBank. EA, Eastern Asia, viruses came from China, Laos, Vietnam, Japan, Korea, and so on; H5, came from H5 subtype AIVs; H9/SH98, came from H9 subtype AIVs; SH, Shanghai. Virus like/lineages are shown at right.

**Figure 7:**
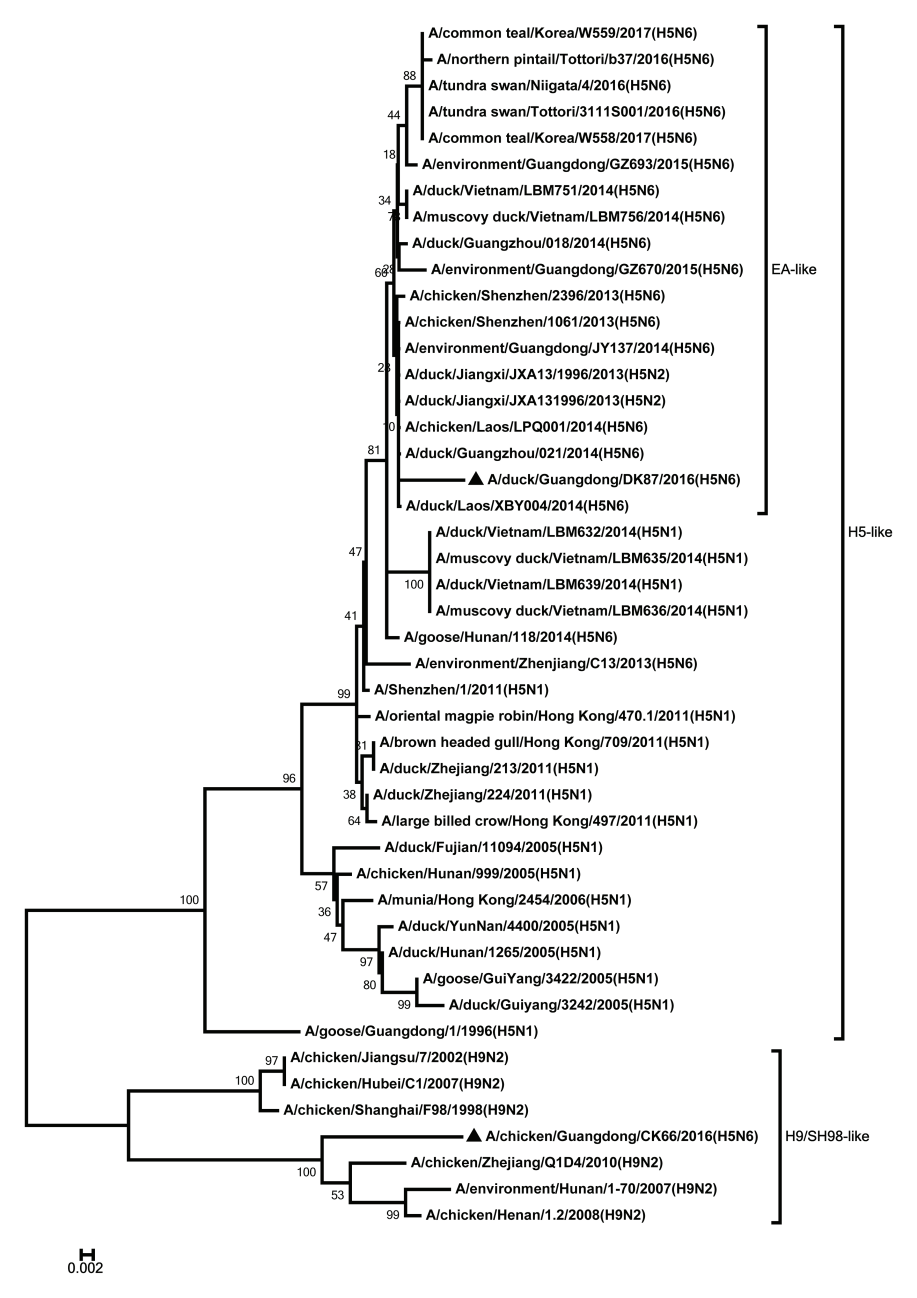
Phylogenetic analysis of M. M: nt 1–733. Black triangles indicate viruses characterized in this study; other viral sequences were downloaded from GenBank. EA, Eastern Asia, viruses came from China, Laos, Vietnam, Japan, Korea, and so on; H5, came from H5 subtype AIVs; H9/SH98, came from H9 subtype AIVs; SH, Shanghai. Virus like/lineages are shown at right.

**Figure 8:**
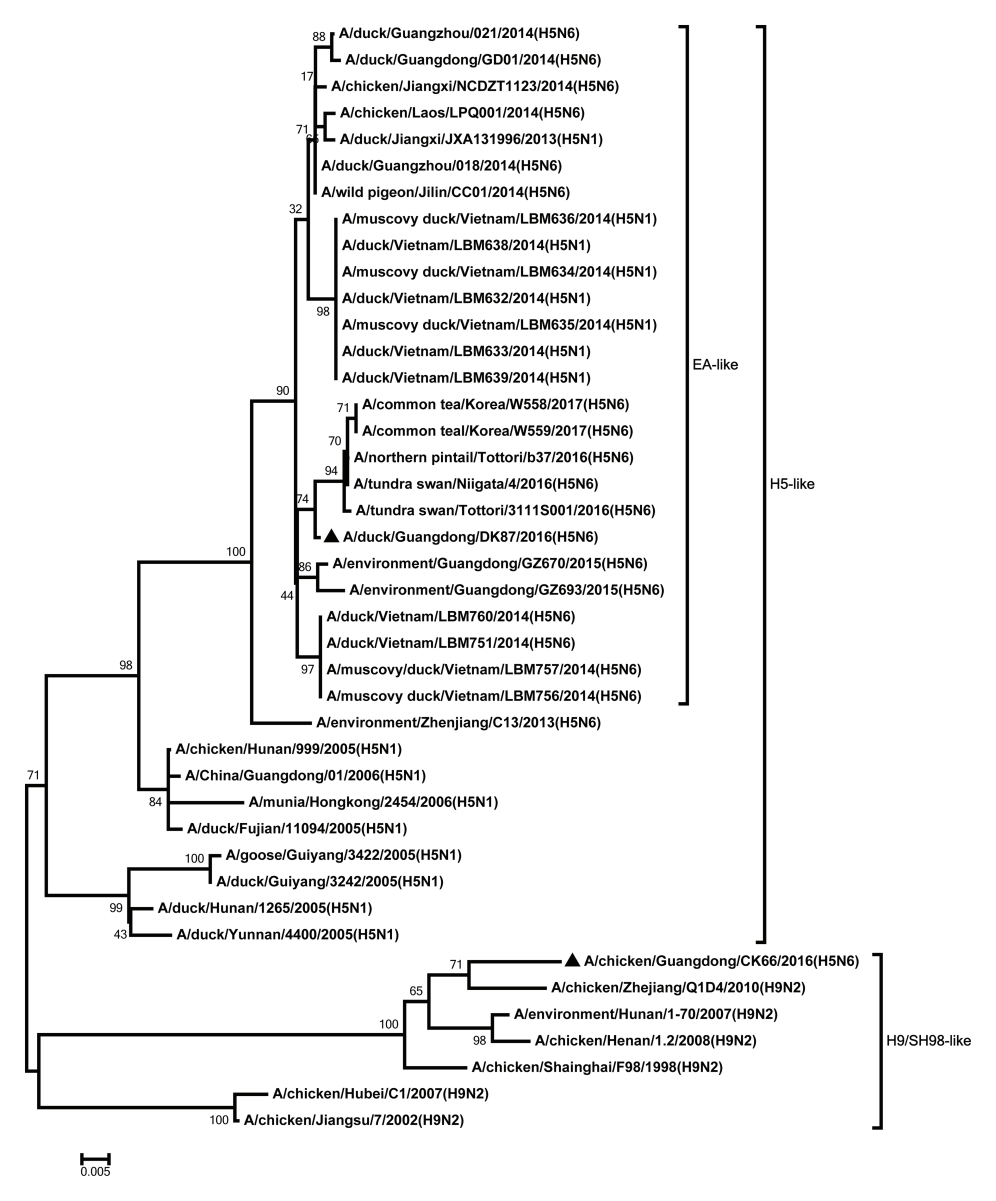
Phylogenetic analysis of NS. NS: nt 1–645. Black triangles indicate viruses characterized in this study; other viral sequences were downloaded from GenBank. EA, Eastern Asia, viruses came from China, Laos, Vietnam, Japan, Korea, and so on; H5, came from H5 subtype AIVs; H9/SH98, came from H9 subtype AIVs; SH, Shanghai. Virus like/lineages are shown at right.

All internal genes of the DK87 were derived from H5 AIV/EA-like lineage (Figures 3–8). The PB2 gene of CK66 was derived from H5 AIV/EA-like lineage (Figure 3), but the other internal genes of CK66 were derived from the H9N2 AIV/SH98-like lineage, which suggesting that CK66 was a double reassortment virus of H5N6 AIVs and H9N2 AIVs (Figures 4–8). The two viruses possessed an E residue at position 627 and D residue at position 701 in PB2 protein (Supplementary Table S1). Mutations of D92E, L103F, and I106M in the NS1 protein were observed in the DK87 virus, but these mutations did not appear in the CK66 virus. Deletion in the NS1 protein at positions 80–84 was presented in the DK87 virus. The PDZ binding motif ESEV of NS1 typically seen in avian viruses was observed in DK87, but this motif was not present in CK66 (Supplementary Table S1). Additionally, substitutes of M1 at N30D, I43M, and T215A were seen in these viruses (Supplementary Table S1). Mutation S31N/G of M2 was seen in CK66 virus (Supplementary Table S1). Other mutations of PA, PB1, and NP proteins are shown in Supplementary Table S1.

### Pathogenicity and Shedding of the Two H5N6 HPAIVs in Ducks

To assess the pathogenicity of the two viruses in ducks, each duck was inoculated intranasally with 10^7^ EID_50_ of DK87 or CK66 viruses in a 0.2ml. All ducks in the inoculated groups were observed for 14days or until they died due to virus infection.

During the experimental period, no dramatic symptoms were observed in ducks. All ducks in the two inoculated groups survived until the experiment finished and seroconverted at 14 DPI (except for one duck inoculated with the DK87 virus who died at 13 DPI). At 12 HPI, the DK87 virus was detected in eight organs with mean viral loads ranging from 1.58 to 2.92 log_10_ EID_50_/g (Table 1). The CK66 virus was only found in the three organs with mean viral loads of 1.75–2.75 log_10_ EID_50_/g. At 3 DPI, the DK87 virus could replicate well in all detected tissues causing systemic infection in ducks. Among these organs, the lung, spleen, kidney, and cloacal bursa were the preferential tissues for viral growth with the mean viral loads ranging from 4.16 to 4.75 log_10_ EID_50_/g. The mean titers of the DK87 virus in other organs were 2.58–3.58 log_10_ EID_50_/g. However, the CK66 virus replicated lower than the DK87 virus in all detected organs, with mean titers were 1.5–2.75 log_10_ EID_50_/g. At 5 DPI, the mean titers of the DK87 virus in detected organs were 3.17–5 log_10_ EID_50_/g, and the mean titers of the CK66 virus were 1.5–2.33 log_10_ EID_50_/g (Table 1). The replication of the duck-origin DK87 virus in some organs was significantly higher than those of the chicken-origin CK66 virus at 5 DPI (*p*<0.05, *p*<0.01, and *p*<0.001). Therefore, our results suggested that the chicken-origin reassortant virus CK66 replicated in multiple organs of ducks without prior adaptation but its viral titers were lower than the duck-origin virus DK87 in some organs.

**Table 1:** Replication of the two H5N6 avian influenza viruses (AIVs) in ducks after intranasal inoculation.

Strains	Time	Virus replication in organs (log_10_EID_50_/g)^a^								
		Lung	Liver	Spleen	Kidney	Brain	Trachea	Pancreas	Intestine	Cloacal Bursa
DK87	12 HPI	2.67 ± 0.52 (3/3)	<1.5 (0/3)	2.67 ± 1.18 (3/3)	2.92 ± 1.84 (2/3)	1.58 ± 0.14 (1/3)	2 ± 0.66 (2/3)	1.83 ± 0.58 (1/3)	1.83 ± 0.58 (1/3)	2.16 ± 0.58 (2/3)
	3 DPI	4.16 ± 1.77 (3/3)	3.58 ± 2 (2/3)	4.42 ± 2.86 (2/3)	4.75 ± 2.29 (3/3)	3.08 ± 0.8 (3/3)	3.5 ± 1.89 (2/3)	2.58 ± 0.88 (3/3)	3.33 ± 2.18 (2/3)	4.25 ± 2.16 (3/3)
	5 DPI	5 ± 0.43^**^ (3/3)	3.42 ± 0.52 (3/3)	3.58 ± 1^*^ (3/3)	5 ± 1.64^*^ (3/3)	4.08 ± 1.28^*^ (3/3)	4.75 ± 2.04 (3/3)	4.08 ± 2.02 (3/3)	3.17 ± 0.76 (3/3)	3.91 ± 0.29^***^ (3/3)
CK66	12 HPI	1.75 ± 0.43 (1/3)	<1.5 (0/3)	<1.5 (0/3)	<1.5 (0/3)	<1.5 (0/3)	2.5 ± 0.43 (3/3)	2.75 ± 0.25 (3/3)	<1.5 (0/3)	<1.5 (0/3)
	3 DPI	2 ± 0.43 (3/3)	<1.5 (0/3)	1.58 ± 0.14 (1/3)	1.67 ± 0.29 (1/3)	2.17 ± 0.58 (2/3)	2.75 ± 1.09 (2/3)	2.42 ± 0.63 (3/3)	2.17 ± 0.58 (2/3)	2 ± 0.87 (1/3)
	5 DPI	1.58 ± 0.14 (1/3)	2.17 ± 0.58 (2/3)	1.58 ± 0.14 (1/3)	1.75 ± 0.43 (1/3)	<1.5 (0/3)	1.83 ± 0.58 (1/3)	2.33 ± 0.52 (3/3)	1.83 ± 0.58 (1/3)	<1.5 (0/3)

HPI, hour post-inoculation; DPI, day post-inoculation. Viral loads were expressed as means±SD in log_10_EID_50_/g of tissue. Significant differences between mean virus titers in tissues of ducks inoculated with the CK66 virus and the DK87 virus were determined by using Student’s *t*-test.

* *p*<0.05

** *p*<0.01

*** *p*<0.001.

a For statistical analysis, a value of 0.5 was assigned if the virus was not detected from the undiluted sample in three embryonated chicken eggs.

To better understand the shedding of the two H5N6 HPAIVs in the inoculated ducks, oropharyngeal and cloacal swabs were collected at 3, 5, 7, 9, 11, and 13 DPI. The shedding of the DK87 virus was monitored in oropharyngeal swabs within 5 DPI and cloacal swabs within 7 DPI (Table 2). The CK66 was monitored in both oropharyngeal and cloacal swabs within 7 DPI (Table 2). That is, the two H5N6 HPAIVs could be delivered throughout the respiratory tract and digestive tract.

**Table 2:** Viral shedding in cloacal and oropharyngeal swabs.

Strain	Infection sample	3 DPI		5 DPI		7 DPI		9 DPI		11 DPI		13 DPI	
		T	C	T	C	T	C	T	C	T	C	T	C
DK87	Inoculated^a^	5/11	5/11	2/8	2/8	0/5	1/5	0/5	0/5	0/5	0/5	0/4	0/4
	Contacted^b^	2/5	2/5	1/5	1/5	1/5	1/5	1/5	0/5	0/5	0/5	0/4	0/4
CK66	Inoculated	2/11	2/11	2/8	2/8	1/5	2/5	0/5	0/5	0/5	0/5	0/4	0/4
	Contacted	2/5	0/5	1/5	3/5	1/5	3/5	0/5	1/5	0/4	0/4	0/4	0/4

DPI, day post-inoculation.

a Ducks inoculated with virus.

b Naive contact duck housed with those inoculated.

### Transmission of Two H5N6 HPAIVs in Ducks

To determine whether the two H5N6 HPAIVs transmit between ducks, five additional ducks (as contacted group) were inoculated intranasally with 0.2ml PBS and then housed with the inoculated ducks of each group.

One death was seen in the DK87-contacted group and the CK66-contacted group during the test time, respectively (Table 3). No contacted ducks exposed to the DK87 or CK66 virus showed obvious clinical signs. The shedding of the DK87 virus was detected from oropharyngeal swabs within 9 DPI and cloacal swabs within 7 DPI (Table 2). Shedding of the CK66 virus could be tested from oropharyngeal swabs within 7 DPI and cloacal swabs within 9 DPI (Table 2). In summary, these results indicated that two H5N6 HPAIVs could horizontally transmit in native ducks by direct contact.

**Table 3:** Replication of the two H5N6 AIVs in the tissues of dead contact ducks.

Strains	Dead time	Virus replication in organs (log_10_EID_50_/g)^a^					
		Lung	Spleen	Kidney	Brain	Trachea	Pancreas
DK87-contacted duck	12 DPI	2.25	1.75	1.75	2.25	2.25	2.5
CK66-contacted duck	10 DPI	1.75	2.25	2.5	1.75	3	2.5

Viral loads were expressed as means ± SD in log_10_EID_50_/g of tissue.

a For statistical analysis, a value of 0.5 was assigned if the virus was not detected from the undiluted sample in three embryonated chicken eggs.

### Expression of PRRs in the Lungs of Ducks Infected With the Two H5N6 HPAIVs

To identify the mRNA expression of PRRs in the lungs of inoculated ducks, we obtained relative quantitative level of gene expression of RIG-I, TLR3, MDA5, and TLR7 using quantitative reverse transcription PCR (qRT-PCR).

**Figure 9:**
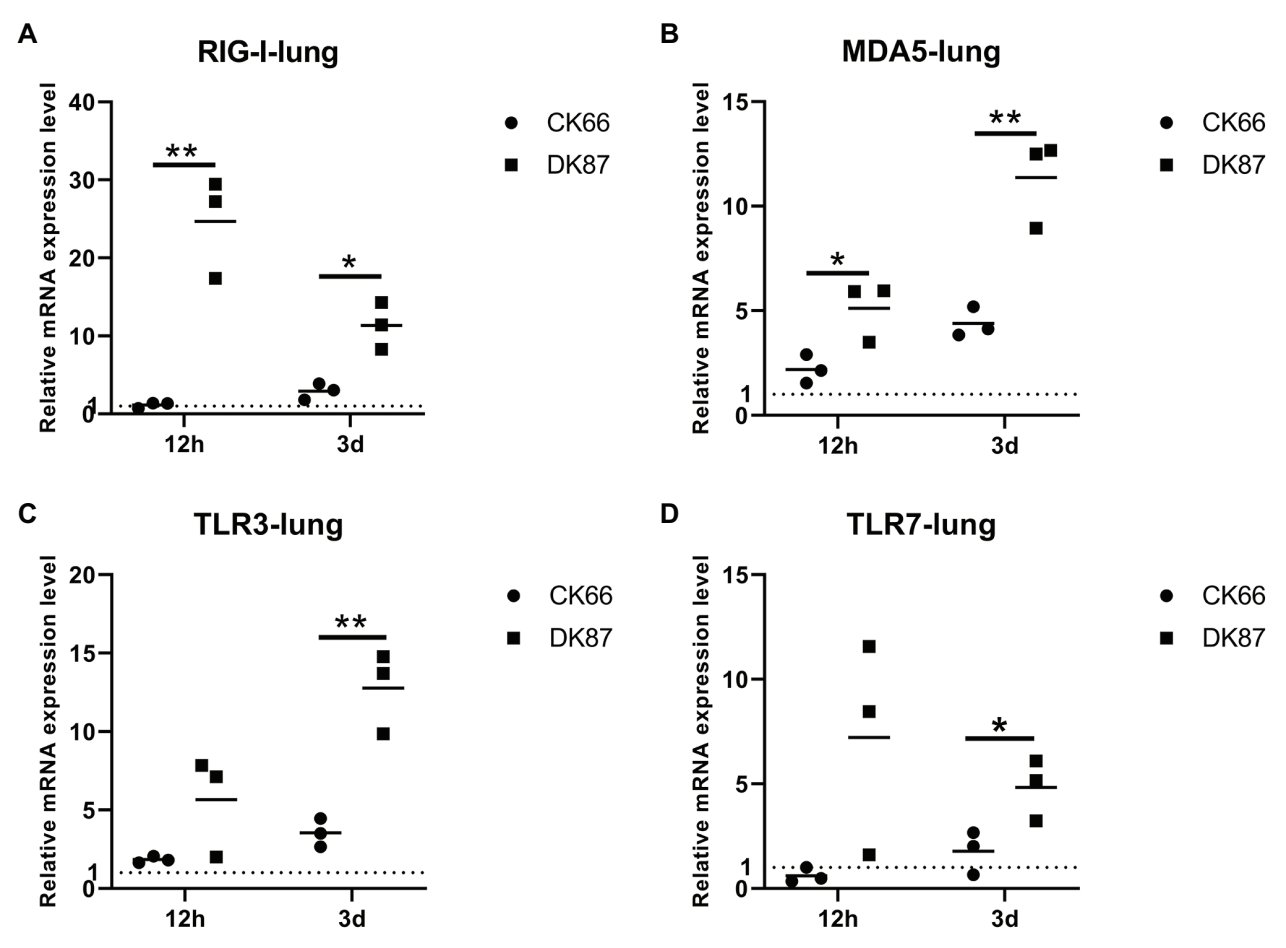
Relative expression of pattern recognition receptors (PRRs) in the lungs of ducks inoculated with DK87 or CK66 viruses **(A–D)**. RNA was extracted from lung of ducks at 12 HPI and 3 DPI inoculated with DK87 or CK66 viruses. The mRNA expression levels were analyzed by quantitative PCR (qPCR) and normalized to the housekeeping gene β-actin. Fold-induction compared to mock-treated ducks is shown for **(A)** RIG-I, **(B)** MDA5, **(C)** TLR3, and **(D)** TLR7. Each dot represents one duck. Dotted line represents the expression of PRRs in the lungs of mock-treated ducks. Values are presented as mean ± SD. Significant differences between mean expression levels in the lungs of ducks inoculated with the DK87 virus and CK66 virus were analyzed by using Student’s *t*-test (^*^p<0.05 and ^**^p<0.01).

Changes in the expression of RIG-I, TLR3, MDA5, and TLR7 were observed in two viruses-inoculated ducks. RIG-I showed the greatest expression among those genes in response to DK87 virus at 12 HPI and 3 DPI (24.68- and 11.33-fold upregulation, respectively). The expression of RIG-I in the CK66-inoculated ducks was slightly increased at 12 HPI and 3 DPI (1.15- and 2.91-fold, respectively; Figure 9A). The expression of TLR3 in the DK87-inoculated ducks was upregulated by 5.65-fold at 12 HPI and 12.78-fold at 3 DPI. In the CK66-inoculated ducks, the expression was upregulated by 1.83-fold at 12 HPI and 2.19-fold at 3 DPI (Figure 9C). The expression of MDA5 in two viruses-inoculated ducks was increased at 12 HPI and 3 DPI, and the fold change in the DK87-inoculated ducks (5.11- and 11.36-fold, respectively) was significantly higher than that in the CK66-inoculated ducks (2.19- and 4.39-fold, respectively; *p*<0.05; Figure 9B). The expression of TLR7 in response to DK87 virus was upregulated at 12 HPI (7.21-fold) and 3 DPI (4.82-fold; Figure 9D). However, the expression of TLR7 in response to CK66 virus was downregulated (0.61-fold) at 12 HPI and slightly increased at 3 DPI (1.78-fold). These data indicated that the expression of the PRRs in response to two viruses was increased at 3 DPI, and the expression of the DK87-inoculated ducks was significantly higher than those of the CK66-inoculated ducks (*p*<0.05 or *p*<0.01).

### Expression of IFN β, IFN α, and Mx-1 in the Lungs of Ducks Infected With the Two H5N6 HPAIVs

**Figure 10:**
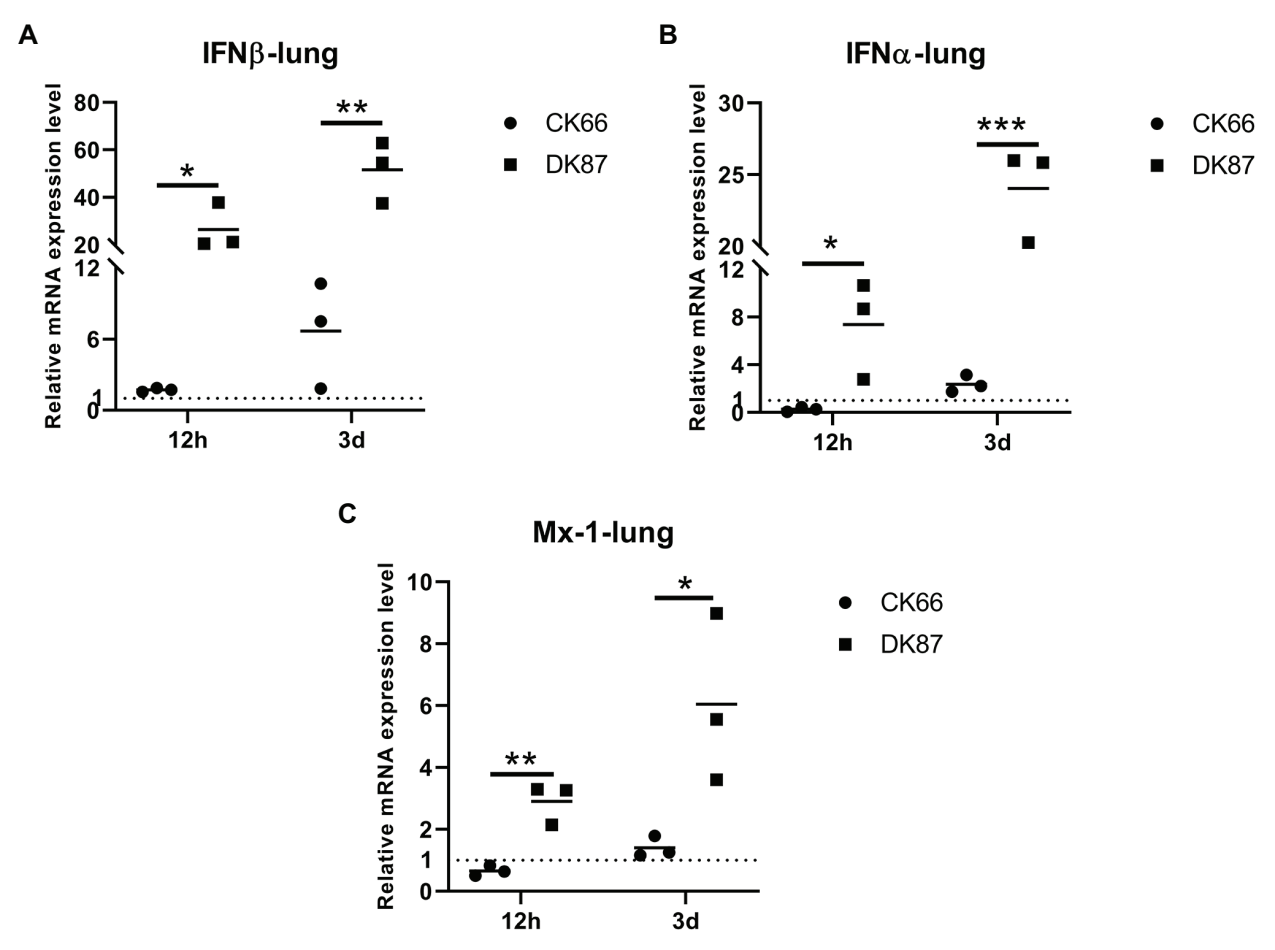
Relative expression of IFNs and Mx-1 in the lungs of ducks inoculated with DK87 or CK66 viruses **(A–C)**. RNA was extracted from lung of ducks at 12 HPI and 3 DPI inoculated with DK87 or CK66 viruses. The mRNA expression levels were analyzed by qPCR and normalized to the housekeeping gene β-actin. Fold-induction compared to mock-treated ducks is shown for **(A)** IFN β, **(B)** IFN α, and **(C)** Mx-1. Each dot represents one duck. Dotted line represents the expression of IFNs and Mx-1 in the lungs of mock-treated ducks. Values are presented as mean ± SD. Significant differences between mean expression levels in the lungs of ducks inoculated with the DK87 virus and CK66 virus were analyzed by using Student’s *t*-test (^*^p<0.05, ^**^p<0.01, and ^***^p<0.001).

Significant upregulation of the IFN β, IFN α, and Mx-1 genes was detected in the lungs of DK87-inoculated ducks vs. CK66-inoculated ducks. Robust expression of IFN β was observed in DK87-inoculated ducks at 12 HPI and 3 DPI (26.43- and 51.62-fold, respectively). This is in response to the CK66 virus that was upregulated by 1.71- and 6.66-fold, respectively (Figure 10A). The expression of IFN α in the DK87-inoculated ducks was increased at 12 HPI and 3 DPI with a change of 7.36- and 24.02-fold, respectively. The expression of this gene in the CK66-inoculated ducks was decreased with a change of 0.26-fold at 12 HPI and slightly increased with a change of 2.71-fold at 3 DPI (Figure 10B). The expression of Mx-1 in CK66-inoculated ducks was downregulated at 12 HPI (0.65-fold), in contrast to the DK87-inoculated ducks (2.90-fold). The expression of Mx-1 induced by two viruses was increased at 3 DPI with a fold change of 1.40-fold (CK66) and 6.50-fold (DK87), respectively (Figure 10C). Thus, vs. the CK66-inoculated ducks, there was a significantly higher expression of IFN β (*p*<0.01), IFN α (*p*<0.001), and Mx-1 (*p*<0.05) in the DK87-inoculated ducks at 3 DPI.

### Expression of IL-6 and IL-8 in the Lungs of Ducks Infected With the Two H5N6 HPAIVs

**Figure 11:**
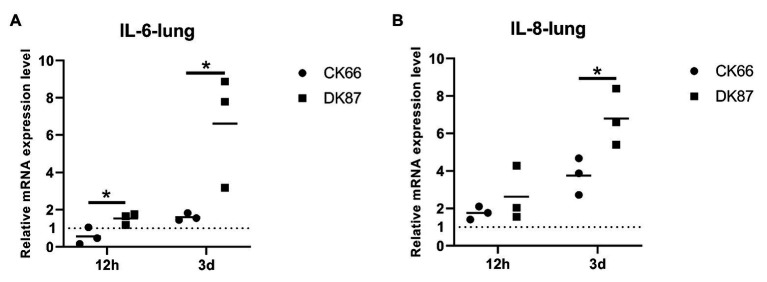
Relative expression of IL-6 and IL-8 in the lungs of ducks inoculated with DK87 or CK66 viruses **(A,B)**. RNA was extracted from lung of ducks at 12 HPI and 3 DPI inoculated with DK87 or CK66 viruses. The mRNA expression levels were analyzed by qPCR and normalized to the housekeeping gene β-actin. Fold-induction compared to mock-treated ducks is shown for **(A)** IL-6 and **(B)** IL-8. Each dot represents one duck. Dotted line represents the expression of IL-6 and IL-8 in the lungs of mock-treated ducks. Values are presented as mean ± SD. Significant differences between mean expression levels in the lungs of ducks inoculated with the DK87 virus and CK66 virus were analyzed by using Student’s *t*-test (^*^p<0.05).

Next, the mRNA expression of IL-6 and IL-8 was measured to evaluate the induction of pro-inflammatory cytokines, and chemokines following the H5N6 AIVs infection. In the lungs of DK87-inoculated ducks, the expression of IL-6 was increased by 1.60-fold at 12 HPI and 6.61-fold at 3 DPI, respectively. In contrast, the lungs of CK66-inoculated ducks had IL-6 expression that was downregulated at 12 HPI and slightly upregulated at 3 DPI with a fold change of 0.56- and 1.52-fold, respectively (Figure 11A). The expression of IL-8 was increased after infection with two H5N6 viruses with a change of 2.62–6.79-fold (DK87) and 1.75–3.75-fold (CK66), respectively (Figure 11B). Therefore, our results suggested that the expression of IL-6 (*p*<0.05) and IL-8 (*p*<0.05) in the DK87-inoculated ducks was significantly higher than those of the CK66-inoculated ducks at 3 DPI.

## Discussion

Since 2014, clade 2.3.4.4 H5N6 HPAIV has been circulating in poultry in Southern China. It has since spread to Laos, Vietnam, Japan, Korea, etc. (Wong et al., 2015; Kwon et al., 2017; Takemae et al., 2017). Duck-origin LPAIVs and/or chicken-origin H9N2/H7N9 AIVs have donated internal genes to local epidemic H5N6 AIVs (Bi et al., 2016; Sun et al., 2018). A genetic analysis showed that the HA genes of our H5N6 HPAIVs fell into clade 2.3.4.4 and were clustered into H5 AIV/EA-like. The NA genes of them were clustered into Eurasian lineage and had close relationship with those of H5N6 viruses circulating in China, Laos, Vietnam, Japan, and Korea. All internal genes of DK87 were derived from H5 AIV/EA-like. The PB2 gene of CK66 was derived from H5 AIV/EA-like, but other internal genes (PB1, PA, NP, M, and NS) of CK66 were derived from H9N2 AIV/SH98-like, which suggested that the CK66 virus was a double reassortment virus of H5N6 AIVs and H9N2 AIVs. These results indicate that clade 2.3.4.4 H5N6 AIVs have continually evolved and recombined with the H9N2 viruses circulating in Southern China. Moreover, some internal genes of the H5N6 viruses that infected human originated from chicken-origin H9N2 AIVs (Zhang et al., 2016). Waterfowl and wild birds are natural hosts for various AIV subtypes, and this contributes to the geographical spread of AIVs and enhances the risk of infected domestic poultry and the mammals including humans (Kim et al., 2009). Therefore, we should understand the genetic and pathogenic variations of clade 2.3.4.4 H5N6 AIVs to prevent outbreaks of this disease.

The amino acid substitutions in the important region of proteins of the AIVs may alter the host adaptation, virulence, tissue tropism, and infectivity (Pantin-Jackwood, 2017). The two H5N6 avian viruses in our study were HPAIVs with multiple basic amino acids at the HA cleavage site. The receptor-binding site at the 226–228 (H3 numbering) motif of the two viruses suggested that they preferred to bind to avian-like (α2–3-galactose sialic acids) receptors. However, mutations of S128P, S137A, and S158N in the HA of DK87 virus and mutations of S137A, S158N, and T160A in the HA of CK66 virus suggested that our viruses may tend to bind to human-like (α 2–6-galactose sialic acids) receptors.

Deletions in the NA stalk region (11 amino acids in N6, positions 58–68) increased the adaptation to poultry and virulence to mammals (Zhou et al., 2009), which were present in our viruses. Our viruses did not have H274Y (N2 numbering) mutations in NA, which have been reported to reduce susceptibility to NA inhibitors (Hurt et al., 2009). Some mutations of internal genes also affect the pathogenicity of AIVs (Hurt et al., 2009). Mutations of E627K and D701N in the PB2 protein normally appeared in mammal-adapted AIVs (Li et al., 2005; Gabriel et al., 2013), but were not present in our viruses, which suggested that they were avian-origin strains. Mutation of N383D in the PA-arch domain was seen in our viruses, and H5N1 AIVs with this mutation killed ducks (Song et al., 2011). Mutation of M105V in the NP protein was observed in CK66 virus and was associated with the pathogenicity of H5N1 AIVs in chickens (Tada et al., 2011). The NS1 protein served multiple functions in the replication and virulence of AIVs (Pereira et al., 2018). A previous study showed that the H5N1 strain inserted five amino acids (80–84) in the NS1 protein and decreased its pathogenicity in mallard ducks (Li et al., 2014). In addition, mutations of P42S, L103F, and I106M in the NS1 protein inhibited the IFN-relative immune response and enhanced the virulence of H5N1 in mice (Twu et al., 2007; Jiao et al., 2008). All of the changes in NS1 mentioned above were observed in DK87 virus, but this was not seen in the CK66 virus. Mutations of M1 at I43M enhanced the virulence of H5N1 AIVs in ducks and chickens and were seen in both viruses (Nao et al., 2015). The S31N/G mutation of M2 was seen in the CK66 virus. This increased resistance to amantadine and rimantadine (Cheung et al., 2006). Overall, different molecular characteristics of the two viruses may result in their different pathogenicity.

In contrast to ducks, chickens are highly susceptible to GS/GD lineage H5 subtype HPAIVs with multiple organs failure associated with systemic virus replication and high mortality rates (Pantin-Jackwood and Swayne, 2009). A recent study showed that chickens are highly susceptible to clade 2.3.4.4 H5N6 HPAIVs with severe clinical signs and 100% mortality rates; ducks inoculated with some strains showed wryneck and neurologic clinical signs with 50–100% mortality rates (Uchida et al., 2019).

In our study, the chicken-origin reassortant virus CK66 replicated in multiple organs of ducks without prior adaptation, but viral titers of the DK87-inoculated ducks in some organs were significantly higher than those of the CK66-inoculated ducks at 5 DPI (*p*<0.05, *p*<0.01, and *p*<0.001). The transmission of AIVs between dabbling ducks is considered though the fecal-oral route. However, other birds may be infected if exposed to virus-contaminated water. Additionally, Bertran et al. (2017) demonstrated that airborne transmission of H5N1 HPAIVs can occur from inoculated chickens and ducks to those non-inoculated, but the exposed native ducks showed no clinical signs and death. Our viruses were transmitted from experimentally infected ducks to co-housed ducks by direct contact and caused one death pet contacted group. Additionally, the possible reason why these dead contacted ducks seems no significantly different virus titers between DK87-gruop and CK66-gruop are as follows: (1) the infectious dose of viruses was different between the experimental infection and natural contact and (2) the individual differences of ducks.

The pathogenicity of AIVs is also affected by the host immune response. Upon AIV infection, the nucleic acids of AIVs were recognized by host PRRs (RIG-I, MDA5, TLR3, and TLR7), resulting in the production of antiviral cytokines (interferon IFN α/β), interferon-induced protein (Mx-1), pro-inflammatory cytokines (IL-6), and chemokines (IL-8; Iwasaki and Pillai, 2014; Pantin-Jackwood, 2016). Duck RIG-I expressed in DF-1 cells increased the IFN β response to reduce the replication of H5N1 AIVs BC200 and VN1203 (Barber et al., 2010). Significant expression of RIG-I was upregulated in the lungs of ducks infected with DK87 virus at 12 HPI (*p*<0.01) and 3 DPI (*p*<0.05) vs. those infected with the CK66 virus. Interestingly, the expression of RIG-I in the lungs and spleens of ducks was lower than that induced by the attenuated strain (H5N1) with PA T515A mutant, although no significant differences in the viral replication was seen in ducks between the parent and mutated viruses (Fleming-Canepa et al., 2019). Similarly, overexpression of duck MDA5 in DEF inhibited the replication of H5N1 virus DK212 (Wei et al., 2014). Moreover, there was a significantly higher expression of IFN β (*p*<0.01), IFN α (*p*<0.001), and Mx-1 (*p*<0.05) induced in the lungs of DK87-inoculated ducks at 3 DPI vs. those of CK66-inoculated ducks. However, protein NS1 of the influenza viruses inhibited the production of IFN β by directly targeting the RIG-I pathway (García-Sastre et al., 1998; Rajsbaum et al., 2012). Wei et al. (2014) found that the MDA5-mediated pathway was inhibited by the NS1 of H5N1 HPAIV. Thus, one possible that the expression of IFN β lower in lungs of CK66-inoculated ducks was the NS1 protein of CK66 virus interferes with the viral activation of the IFN pathway more efficiently than the NS1 of DK87 virus. Additionally, a previous study showed that the D4AT (H5N1) strain with C-terminal ESEV motif of NS1 induced significantly higher expression of IFNs in the lungs or spleens of ducks at 1 DPI vs. the VN1203 (H5N1) strain that lacked this motif; we found similar results (Saito et al., 2018). Hypercytokinemia would enhance the pathogenicity of H5N1 viruses in chickens. IL-6 was rapidly upregulated in chickens infected with the two H5N1 HPAIVs (A/Muscovy duck/Vietnam/453/2004) and (A/Duck/Indramayu/BBVW/109/2006), but not infected ducks (Burggraaf et al., 2014). Similarly, IL-6 and IL-8 were highly upregulated in chickens infected with (A/turkey/England/50–92/91, H5N1-tyEng91) or (A/turkey/Turkey/1/05, H5N1-tyTR05), but these cytokines were unchanged in infected ducks (Kuchipudi et al., 2014). Previous studies have showed that the expression of IL-6 was associated with the pathogen damage to the organs of host and the replication of virus in organs (Karpala et al., 2011; Wang et al., 2014). Here, moderated expression of IL-6 and IL-8 was seen in the two groups of inoculated ducks at 12 HPI and 3 DPI, but significantly higher expression was observed in the DK87-inoculated ducks at 3 DPI (*p*<0.05). Overall, the expression of the immune-associated genes of the ducks may be related to the replication of AIVs and the expression of cytokines triggered by AIVs might contribute the pathogen damage to host, resulting in enhancing the pathogenicity to ducks.

## Data Availability Statement

The datasets presented in this study can be found in online repositories. The names of the repository/repositories and accession number(s) can be found in the article/Supplementary Material.

## Ethics Statement

The animal study was reviewed and approved by the experimental animal administration and Ethics Committee of South China Agricultural University.

## Author Contributions

JH and PJ designed this study, performed the experiments, and drafted the manuscript. SW, WW, YL, HZ, ZY, and QX assisted with animal experiment. JH, PJ, and ML participated in writing the discussion. All authors have read and approved the final manuscript.

### Conflict of Interest

The authors declare that the research was conducted in the absence of any commercial or financial relationships that could be construed as a potential conflict of interest.
