# Supplementary material for: The Biological Characteristics of Novel H5N6 Highly Pathogenic Avian Influenza Virus and Its Pathogenesis in Ducks
Source: Front Microbiol. 2021 Jan 26;12:628545. doi: 10.3389/fmicb.2021.628545 (PMC7874018; doi:10.3389/fmicb.2021.628545)
Supplement: Supplementary file 1 [file Data_Sheet_1.pdf]

Supplementary materials

Table S1. Molecular analysis of the two H5N6 HPAIVs isolated from LPMs

Viral proteins	Phenotypic effect	Mutations or molecular feature	DK87	CK66
HA	Cleavage site Characteristic of HPAIVs	Cleavage sites	PLKERRRK R/GLF	PLRERRRK R/GLF
	Receptor binding sites (H3 numbering) [a]	S128P	P	L
		S137A	A	A
		S158N	N	N
		T160A	S	A
		Q226L	Q	Q
		S227N	Q	S
		G228S	G	G
NA	Increased virulence in mice (N6 numbering) [b]	Stalk deletion	Yes	Yes
	Reduced susceptibility to NA inhibitors[c]	V116A	V	V
		E119D/G	E	E
		H274Y	H	H
PB2 [d]	Mammalian adaptation	K389R	R	K
		M535L	L	M
		A588V	A	V
		V598T/I	V	T
		E627K	E	E
		D701N	D	D
PA [d]	Decreased polymerase activity in mammalian cell line	A37S	A	S
	Increased polymerase activity in avian cell lines	N383D	D	D
PB1 [d]	Enhanced virulence in mice	D622G	G	G
NP [d]	Increases virulence in chickens	M105V	I	V
NS1 [d]	Adaptation to mice	G34S	G	S
	Increased virulence and decreased antiviral response in mice	P42S	S	S
	Increases virulence in chickens and mice	80-84 deletion	Yes	No
	Increased virulence in mice	D92E	E	D

	and chickens			
	Increased virulence in mice	ESEV(PDZ domain)	Yes	No
	Increased virulence in mice	L103F	F	L
		I106M	M	I
M1 [d]	Increased virulence in mice, chickens and ducks	I43M	M	M
	Increased virulence in mice	N30D	D	D
		T215A	A	A
M2 [d]	Increased resistance to amantadine and rimantadine	S31N/G	S	N

Abbreviations: a: Mutation/location H3 numbering relative to A/Aichi/2/1968(H3N2). b: The N6 numbering system is used. c: Mutation/location N2 numbering relative to A/Aichi/2/1968(H3N2). d: Mutation/motifs are numbered according to alignments with A/Goose/Guangdong/1/1996(H5N1).

Table S2. The potential glycosylation site in HA and NA proteins of the two H5N6 HPAIVs.

Genes	Position	Viruses	
		DK87	CK66
HA [a]	26	NNS	NNS
	27	NST	NST
	39	NVT	NVT
	181	NNT	NNT
	302	NSS	NSS
	499	NGT	NGT
	558	NGS	NGS
NA [b]	51	NET	NET
	54	NPT	NQT
	70	NIT	NIT
	86	-[c]	NLT
	146	NGT	NGT
	201	NAS	NAS

Abbreviations: a: The potential N-glycosylation sites at positions of HA protein was numbering according to A/goose/Guangdong/1/1996 (H5N1); b: The potential N-glycosylation sites at positions of NA protein was numbering according to A/duck/Guangdong/s3073/2010(H6N6) and A/duck/Fujian/3242/2007(H6N6); c: The potential N-glycosylation sites at position is absent.

Table S3. Accession numbers of all sequences used in Figure 1-8.

Strains	Genebank code of reference sequences							
	**PB2**	**PB1**	**PA**	**HA**	**NP**	**NA**	**M**	**NS**
A/northern pintail/Tottori/b37/2016(H5N6)	LC200414	LC200415	LC200416	LC200417	LC200419	LC200419	LC200420	LC200421
A/tundra swan/Niigata/4/2016(H5N6)	LC318901	LC318902	LC318903	LC318904	LC318905	LC318906	LC318907	LC318908
A/tundra swan/Tottori/3111S001/2016(H5N6)	LC274915	LC274916	LC274917	LC274918	LC274919	LC274920	LC274921	LC274922
A/common teal/Korea/W558/2017(H5N6)	KY576130	KY576128	KY576126	KY576116		KY576120	KY576118	
A/common teal/Korea/W559/2017(H5N6)	KY576131	KY576129	KY576127	KY576117		KY576121	KY576119	
A/environment/Guangdong/GZ693/2015(H5N6)	KU852961		KU852953	KU852944		KU852947	KU852962	KU852962
A/duck/Guangzhou/41227/2014(H5N6)				KP765796				
A/duck/Vietnam/LBM751/2014(H5N6)	LC028189	LC028190	LC028191	LC028192	LC028193	LC028194	LC028195	LC028196
A/muscovy duck/Vietnam/LBM756/2014(H5N6)	LC028312	LC028313	LC028314	LC028315	LC028316	LC028317	LC028318	LC028319
A/Guangdong/99710/2014(H5N6)				EPI_ISL_219828				
A/chicken/Shenzhen/2396/2013(H5N6)				KP284989	KP284992			
A/environment/Guangdong/GZ670/2015(H5N6)	KU852961	KU852958	KU852955	KU852946	KU852952	KU852949	KU852964	KU852965
A/Yunnan/14564/2015(H5N6)				EPI_ISL_178262				
A/chicken/Jiangsu/7/2002(H9N2)	FJ384748	FJ384749	FJ384750		FJ384752		FJ384754	FJ384755

Strains	Genebank code of reference sequences							
	PB2	PB1	PA	HA	NP	NA	M	NS
A/chicken/Laos/LPQ001/2014(H5N6)	KM496962	KM496963	KM496964	KM496965	KM496966	KM496967	KM496968	
A/duck/Laos/LPQ002/2014(H5N6)				KM496970		KF735645		
A/environment/Guangdong/40113/2015(H5N6)				EPI_ISL_219808		CY146702		
A/Shenzhen/1/2015(H5N6)				EPI_ISL_205313		KT762441		
A/duck/Guangdong/018/2014(H5N6)				KX094400		CY098808		
A/duck/Eastern China/S0908/2014(H5N6)				KP732643		CY098800		
A/muscovy duck/Vietnam/LBM635/2014(H5N1)	AB979484	AB979485	AB979486	AB979487	AB979488	GU050343	AB979490	
A/muscovy duck/Vietnam/LBM639/2014(H5N1)	AB979508	AB979509	AB979510	AB979511	AB979512	CY089471	AB979514	
A/muscovy duck/Vietnam/LBM631/2014(H5N1)	AB979452			AB979455	AB979456	KP097971		
A/muscovy duck/Vietnam/LBM636/2014(H5N1)		AB979493	AB979494	AB979495	AB979496	CY098746	AB979498	
A/environment/Chongqing/45355/2015(H5N2)				EPI_ISL_219777		CY098753		
A/wild duck/Shandong/628/2011(H5N1)	JX534562			JX534565		CY098725		
A/duck/Jiangsu/m234/2012(H5N2)				JX507355		CY098816		
A/baikal teal/Korea/H52/2014(H5N8)				KJ508961		CY091637		
A/duck/Eastern China/1111/2011(H5N1)				JQ041401		HM172167		
A/environment/Zhenjiang/C13/2013(H5N6)	KJ938655		KJ938657	KJ938658	KJ938659		KJ938661	KJ938662

Strains	Genebank code of reference sequences							
	PB2	PB1	PA	HA	NP	NA	M	NS
A/Hunan/1/2009(H5N1)				CY098723		HM172172		
A/chicken/Sichuan/81/2005(H5N1)				HM172069		HM172171		
A/common magpie/Hong Kong/645/2006(H5N1)				DQ992839		HM172197		
A/Anhui/1/2005(H5N1)				HM172104				
A/chicken/Vietnam/NCVD-20/2007(H5N1)				CY030383		AY818142		
A/muscovy duck/Vietnam/41/2007(H5N1)				CY029679				
A/avian/Hong Kong/1993/2007(H5N1)				GU050341		AY651433		
A/muscovy duck/Vietnam/39/2007(H5N1)				CY029671		CY017656		
A/goose/Yunnan/6193/2006(H5N1)				CY030977		CY017690		
A/environment/Guizhou/2/2009(H5N1)				CY098798		CY014282		
A/duck/Guiyang/3242/2005(H5N1)		DQ992756	EF124759	DQ992756	EF124415	EU146761	EF124155	EF124608
A/duck/Guiyang/3422/2005(H5N1)	DQ992678	EF124005	EF124760	KJ522742	EF124458	DQ095657	EF124156	EF124609
A/chicken/Guiyang/3055/2005(H5N1)				DQ992755		EF619973		
A/chicken/Hunan/999/2005(H5N1)	DQ320844	DQ321305	DQ321239	DQ320910	DQ321107		DQ320976	DQ321173
A/duck/Hunan/139/2005(H5N1)				DQ320903				
A/duck/Hunan/1265/2005(H5N1)	DQ320845	DQ321306	DQ321240	DQ320911	DQ321108	DQ321043	DQ320977	DQ321174

Strains	Genebank code of reference sequences							
	PB2	PB1	PA	HA	NP	NA	M	NS
A/duck/Yunnan/4400/2005(H5N1)	DQ992797			DQ992797	EF124444		EF124142	EF124595
A/goose/Guangxi/345/2005(H5N1)				DQ320896				
A/goose/Guangdong/1/1996/(H5N1)	AF144300	AF144301	AF144302	AF144305			AF144306	
A/environment/Guangdong/HZ057/2013(H6N6)						KT370001		
A/duck/Shantou/1984/2007(H6N6)						CY109764		
A/mallard/Shantou/972/2005(H6N6)						ADG43192.1		
A/duck/Shantou/9350/2006(H6N6)						CY110314		
A/duck/Fujian/958/2006(H6N6)						CY109492		
A/duck/Fujian/3242/2007(H6N6)						CY110126		
A/goose/Guangdong/S4362/2009(H6N6)						KJ200935		
A/environment/Zhenjiang/C13/2013(H5N6)						KJ938660		
A/duck/Guangdong/S3468/2010(H6N6)						KJ200799		
A/duck/Guangdong/S1155/2011(H6N6)						KJ200767		
A/chicken/Guangdong/S1414/2010(H6N6)						KJ200655		
A/duck/Hunan/S4273/2010(H6N6)						KJ200887		
A/duck/Zhejiang/S1134/2011(H6N6)						KJ200911		

Strains	Genebank code of reference sequences							
	PB2	PB1	PA	HA	NP	NA	M	NS
A/duck/Zhejiang/S4204/2010(H6N6)						KJ200919		
A/turkey/Minnesota/957/80(H6N6)						AY207547.1		
A/northern shoveler/Oregon/44336-179/2007(H4N6)						CY076471		
A/blue-winged teal/Texas/Sg-00085/2007(H3N6)						CY078259.1		
A/northern shoveler/Oregon/44336-179/2007(H4N6)						CY076471		
A/blue-winged teal/Texas/Sg-00085/2007(H3N6)						ACX93213.1		
A/northern/pintail/Alberta/265/2007(H4N6)						CY103342.1		
A/mallard/California/1418/2013(H5N6)						CY176975		
A/mallard/California/1500P/2013(H5N6)						CY177427		
A/duck/Zhejiang/224/2011(H5N1)							JN646735	
A/mallard/California/940V/2013(H4N6)						CY177103		
A/duck/Hubei/WH18/2015(H5N6)						KX652136		
A/chicken/Hubei/XG18/2015(H5N6)						KX652137		
A/chicken/Shenzhen/1061/2013(H5N6)					KP286088	KP286087		
A/duck/Guangxi/175D12/2014(H3N6)						KR919741.1		

Strains	Genebank code of reference sequences							
	PB2	PB1	PA	HA	NP	NA	M	NS
A/duck/Laos/XBY004/2014(H5N6)	KM496974	KM496975	KM496976		KM496978	KM496979	KM234810	KM234811
A/chicken/Jiangxi/13202/2014(H10N6)						KP285959	KM496980	KM496981
A/environment/Guangdong/HZ057/2013(H6N6)						KT370001	KM496980	
A/duck/Fujian/11094/2005(H5N1)	DQ992636	EF123962	EF124717		EF124415		EF124113	EF124566
A/duck/Eastern China/008/2008/(H5N5)	GU727650							
A/duck/Eastern/China/108/2008(H5N1)	GU727666							
A/duck/Eastern China/031/2009(H5N5)	GU727658							
A/duck/Guangdong/wy24/2008(H5N5)	CY091640							
A/duck/Guangdong/wy11/2008(H5N5)	CY091627							
A/China/Guangdong/01/2006(H5N1)	DQ835310		DQ835312					
A/chicken/Zhejiang/Q1D4/2010(H9N2)	JN869521	JN869522			JN869524		JN869526	JN869527
A/munia/HongKong/2454/2006(H5N1)	DQ992565	EF123891	EF124646		EF124344		EF124042	EF124495
A/teal/Tottori/1/2016(H5N6)	LC199865							
A/chicken/Vietnam/2015(H5N6)	KY171706.1							
A/goose/Vietnam/2015(H5N6)	KY171691.1							
A/large billed crow/Hong Kong/497/2011(H5N1)							KC436118	

Strains	Genebank code of reference sequences							
	PB2	PB1	PA	HA	NP	NA	M	NS
A/chicken/Hubei/C1/2007 (H9N2)	EU365368	EU365369	EU365370		EU365372		EU365374	EU365375
A/chicken/Shanghai/F98/1998(H9N2)	AY253750	AY253751	AY253752		AY253753		AY253755	AY253756
A/environment/Hunan/1-70/2007(H9N2)	GU474563	GU474564	GU474565		GU474567		GU474569	GU474570
A/chicken/Henan/1.2/2008(H9N2)	ACK43773	ACK43771	ACK43775		ACK43774		ACK43770	ACK43767
A/duck/Vietnam/LBM752/2014(H5N6)		LC028198						
A/muscovy duck/Vietnam/LBM754/2014(H5N6)		LC028206						
A/muscovy duck/Vietnam/LBM755/2014(H5N6)		LC028305						
A/duck/Jiangxi/JXA132023/2013(H5N2)		KM234813						
A/duck/Vietnam/LBM632/2014(H5N1)		AB979461	AB979462		AB979464		AB979466	
A/duck/Vietnam/LBM360c1-4-1/2013(H5N6)		LC010694						
A/China/Guangdong/01/2006(H5N1)		DQ835311						
A/quail/Guangxi/B1/2006(H9N2)		EU086297						
A/whooper swan/Niigata/12/2017(H5N6)		EPI_ISL_2 90311						
A/whooper swan/Niigata/13/2017(H5N6)		LC318926						
A/peregrine falcon/Hokkaido/X7/2016(H5N6)		LC317076						
A/duck/Vietnam/LBM638/2014(H5N1)			AB979502		AB979504			

Strains	Genebank code of reference sequences							
	PB2	PB1	PA	HA	NP	NA	M	NS
A/chicken/Heilongjiang/S7/2014(H5N2)			KX160176.1					
A/chicken/Shenzhen/2464/2013(H5N6)			KP284996					
A/duck/Jiangxi/JXA131996/2013(H5N2)			KM234808		KM234809			
A/whooper swan/Korea/Gangjin/48/2016(H5N6)					KX160174.1			
A/teal/Tottori/2/2016(H5N6)					LC200937			
A/environment/Fujian/FJ1273/2014(H1N2)					KP658061			
A/chicken/Dongguan/3363/2013(H5N6)					KP286110			
A/muscovy duck/Vietnam/LBM634/2014(H5N1)					AB979480			
A/China/GD01/2006(H5N1)					EF124344			DQ835317
A/duck/Vietnam/LBM760/2014(H5N6)								LC028351
A/muscovy/duck/Vietnam/LBM757/2014(H5N6)								LC028327
A/duck/Guangzhou/018/2014(H5N6)							KX094401	
A/environment/Guangdong/JY137/2014(H5N6)							KT370071	
A/duck/Jiangxi/JXA13/1996/2013(H5N2)							KM234810	
A/duck/Guangzhou/021/2014(H5N6)							KX094409	
A/goose/Hunan/118/2014(H5N6)							KX121201	

Strains	Genebank code of reference sequences							
	PB2	PB1	PA	HA	NP	NA	M	NS
A/Shenzhen/1/2011(H5N1)							KC436112	
A/oriental magpie robin/Hong Kong/470.1/2011(H5N1)							KC436114	
A/brown headed gull/Hong Kong/709/2011(H5N1)							KX160172.1	
A/duck/Zhejiang/213/2011(H5N1)							JN646734	
A/duck/Dongguan/2685/2013(H5N6)						KP285007		
